# 

**Published:** 1873-05

**Authors:** 


Erie County Medical Society.—The Erie County Medical Society will hold its regular annual meeting at the rooms of the Buffalo Medical Association, on Tuesday, June 10. It is hoped that this meeting will be largely attended, and that every member will make an effort to be present.

### Books and Pamphlets Received.

The Principles and Practice of Surgery. By Frank Hastings Hamilton, A. M., M. D., LL. D., Professor of the Practice of Surgery, with Operations, and of Clinical Surgery, in Bellevue Hospital Medical College, etc. Second Edition, revised and corrected. New York: Wm. Wood & Co.

Handbook for the Physiological Laboratory. By E. Klein, M. D., Assistant Professor in the Pathological Laboratory of the Brown Institution, London, formerly Private Docent in Histology in the University of Vienna; J. Burdon Sanderson, M. D., F. R. S., Professor of Practical Physiology in University College, London; Michael Foster, M. A., M. D., F. R. S., Fellow of, and Prælector of Physiology in, Trinity College, Cambridge; and T. Lauder Brunton, M. D., D. Sc, Lecturer on Materia Medica in the Medical College of St. Bartholomew's Hospital, London. Edited by J. Burdon-Sanderson. In two Volumes, with three hundred and fifty-three Illustrations. Vol. I., Text. Vol. II, Plates. Philadelphia: Lindsay & Blakiston.

A Handbook of Medical Electricity. By Herbert Tibbits, M. D., L. R. C. P., London, Medical Superintendent of the National Hospital for the Paralyzed and Epileptic, Medical Officer for Electrical treatment to the Hospital for Sick Children, Great Ormond Street. With sixty-four Illustrations. Philadelphia: Lindsay & Blakiston, 8 vo., pp. 164.

Civil Malpractice: A Report presented to the Military Trust Medical Society, at its Fifteenth Semi-Annual Meeting, January 14th, 1873. By M. H. McClelland, M. D. Chicago: W. B. Keen, Cooke & Co. 8 vo., pp. 74. Price $2.00.

Diseases of the Mastoid Process: their Diagnosis, Pathology and Treatment. By Albert H. Buck, M. D. Reprinted from Archives of Ophthalmology and Otology.

The Unconscious Action of the Brain, and Epidemic Delusions. By Dr. W. B. Carpenter, F. R. S., author of "Human Physiology," "The Microscope and its Revelations." Being Part VI. of "Half Hour Recreations in Popular Science." Boston: Estes & Lauriat. Price 25 cents.

Catalogue of the Medical Works published by James Campbell, Boston, Mass.

Annual Report of the New York State Inebriate Asslum at Binghamton, New York, for the year 1872.

Catalogue of American and Foreign Architectural and Scientific Books and Journals published and for sale by A. J. Bicknell & Co., New York.

**THE BUFFALO**

### Medical and Surgical Journal

**PUBLISHED MONTHLY,**

Containing Original Articles, Reports of Medical Societies and Hospitals. Editorial, Reviews, Correspondence, Army News, etc., etc; including the usual variety of Medical Periodical Publications. Specimen copies sent on application.

Address Buffalo Medical & Surgical Journal, Buffalo, N. Y.

TERMS OF SUBSCRIPTION, - - $3.00 PER YEAR, IN ADVANCE.

